# A conversation with Dr. William Gruber, Pfizer Inc.

**DOI:** 10.1017/cts.2022.16

**Published:** 2022-02-10

**Authors:** Kathy Siranosian

**Affiliations:** Clinical Research Forum, Washington DC, USA

## Top 10 Clinical Research Achievement Awards Q & A

*The Clinical Research Forum wishes to acknowledge the incredible scientific achievement and effort of the many colleagues that have led to the successful development of the Pfizer and Moderna COVID-19 vaccines. The rapid development of these vaccines shows what clinical and translational research can accomplish at its best. These are the first RNA-vaccine constructs ever used for a major international vaccine, and the researchers of both vaccines, along with the countless others on the team who supported the effort, are therefore especially to be commended, heralded, and congratulated. This achievement is a demonstration of the research continuum from basic science through translation into clinical research to safe and effective vaccines to protect human health. In 2021, we honored Pfizer and Moderna with the Clinical Research Forum Award for Extraordinary Impact on Health. We begin this interview series with Dr William Gruber, Senior Vice President of Clinical Research and Development from Pfizer, who is responsible for the global clinical development of vaccines and led the Pfizer-BioNTech COVID-19 vaccine effort. This interview has been edited for length and clarity.*

## When did you initially get excited about clinical research?

When I was in medical school, it became clear to me that I wanted to work in a research environment. At that point, I had already started doing some bench work, but my path really opened up when I started working with Paul Glezen, MD, and his team on respiratory syncytial virus (RSV), the most common viral cause of bronchiolitis and pneumonia in children under one year of age and a significant cause of lower respiratory disease in older adults. As a pediatric infectious disease fellow at Baylor College of Medicine, I was looking at the potential to use aerosolized ribavirin to treat bronchiolitis, the disease that’s associated with RSV in infants. I remember coming in for one of my shifts and seeing that there were six kids in the ICU on ventilators. These were infants less than six months of age, and they were so sick and so fragile. All I could think was, “There has to be a better way.” I knew then that I wanted to be involved in clinical research and work on intervention strategies like vaccines. I wanted to be able to do something that could prevent catastrophes from happening, rather than trying to treat a disease that was already in progress.

From that point, I was on a clear path. One of my first goals was to understand better how to intervene against RSV – and it is a worthy target. In terms of public health, RSV is the single largest cause of hospitalization for infants in most of the developed world and is a major cause of infant mortality in low- and middle-income countries (LMICs), as well. In the United States alone, there are between 40,000 and 120,000 children hospitalized with RSV each year. It also accounts for about one-third to half as much respiratory disease as that associated with influenza in adults. We are making progress, though. There are candidate vaccines for both maternal and adult immunization currently in Phase 3 clinical trials.

## Who were the people that inspired you then, early in your career?

Three people immediately come to mind. First, Paul Glezen, MD, as I mentioned above. Then, there was Carol Baker, MD, who is the world’s foremost authority on Group B Streptococcus, the single most important bacterial pathogen for young children in the post-pneumococcal-vaccine age. When I met Carol, she had just come back from sabbatical in Boston where she was doing both clinical and basic laboratory work on a vaccine against Group B Strep, and she was so vibrant and dynamic. She was really a triple threat – a great clinician, a great clinical researcher, and a great basic science researcher – and I said to myself, “I want to be like her.” There is now a Group B Strep vaccine candidate ready to move forward to Phase 3 clinical trials, and I can’t think of anything that would make me more satisfied than to be part of the team that develops a vaccine against something Carol began talking about in the late 1960s. I was also inspired by Ralph Feigin, MD, who was Chair of the Baylor College of Medicine Department of Pediatrics from 1977–2008. His career was dedicated to relieving suffering caused by pediatric infectious disease, and he was probably the most brilliant person I’ve ever encountered in my life. He took an interest in my career and helped me in so many ways. I was really fortunate to be in this particular environment at this time at Baylor, where I was inspired by these people and countless others.

## That brings us to the team science aspect of your work. How are others involved?

I’m glad that you specifically mentioned the team aspect, because often I see myself as primarily being a cheerleader or someone who removes obstacles so a bunch of talented people can do the work that needs to get done. I’m blessed with a great group of folks, some of whom have been working with me since I left Vanderbilt for a career in industry – and that was over 22 years ago. They’re people I trust implicitly and interestingly enough, not all of them followed the usual steps to get here. The vice president of my clinical scientist group, for instance, was tending bar when she was hired to work in the lab. Now, she’s one of the key people on my team. And for the COVID-19 vaccine work, I have to mention the clinical trial participants and their families; sites, investigators, and their dedicated staff; our clinical trial clinical research organization and other partners; various governments and regulatory authorities; and my colleagues at BioNTech and Pfizer. Every one of them is part of the team. I would also like to give a special shout out to the “Polio Pioneers,” who participated as children in 1954 in the field trials of the Salk polio vaccine and then stepped up once again to participate in the COVID-19 vaccine trials. Their commitment to research science and volunteerism is truly remarkable.

## What three pieces of advice do you have for someone who wants to pursue a career in clinical research?

First, find a mentor. Why is that so critical? Because the three people I mentioned above opened up what’s possible. When you’re inexperienced and just starting out, it’s easy to be constrained by the limitations of the past. You need mentors to remind you to think more broadly. The COVID-19 story is a powerful example of that because we basically had to develop a Phase 1, 2, and 3 studies that were seamless throughout, as opposed to conducting it iteratively at each stage. That doesn’t mean we took shortcuts. It means we had to create seamless processes and work directly with regulators in real time. Asking ourselves “What’s possible?” was essential; otherwise, we would not have been able to go from a standing start to an emergency use authorization in nine months. Mentors help you think innovatively so you’re not limited by conventional wisdom.

Second, don’t be arrogant. Don’t be so convinced that you think you know what the future is going to be that you’re completely sold on your own propaganda. You may have a lot of evidence to support your point of view, but you have to recognize that there are going to be both successes and failures. I learned this when I was working with HIV. We had a very ambitious program looking at several different modalities – vaccines that were peptide-based, DNA-based, and vector-based – and we were going full throttle under the assumption that primate data were predictive. Then everything came to a crashing halt when it didn’t work like we thought it would. Finding that enabling technology for HIV hasn’t been easy and thankfully, there are many people still trying to develop it.

Which leads right into my third piece of advice: Be persistent. In clinical research, achievement can take decades. We are continually building on the accomplishments – and failures – of others. You need to be open to new ideas and be flexible enough that you don’t miss important information, and you also need to believe in yourself and keep going. Persistence is absolutely essential.

## Outside of clinical research, what other activities do you enjoy? How do these activities impact your work?

I love nature – maybe that comes with having an interest in biology. My parents went to the University of Colorado, and I grew up hiking and climbing there. Now, I live in upstate New York and my backyard abuts the Appalachian Trail. Whenever I want to recharge, get reinspired, or work through a problem, I try to get outside and go hiking. I find it incredibly relaxing and soothing. There are a few outcroppings – I call them “my rocks” – that I hike to so I can just sit and think. Beyond that, any free time I have I spend mostly with my wife, our four sons, and their families. It’s not always easy to find downtime, but it is so valuable.

